# Persistent Jaundice and Splenomegaly in Young Persons

**Published:** 1908-10-31

**Authors:** 


### MEDICINE.

### PERSISTENT JAUNDICE AND SPLENOMEGALY IN YOUNG PERSONS.

THERE is a distinct clinical condition that comes under observation from time to time, the most obvious characteristics of which are:—(1) Chronic jaundice of almost indefinite duration—starting in early life, but persisting for many years, some patients surviving middle age without ever losing a jaundice which began when they were not yet out of their teens. (2) More or less marked anæmia. (3) Decided, and sometimes considerable, hypertrophy of the spleen, with enlargement of the liver in less degree. (4) A distinct tendency for the disease to affect several members of the same family—parent and child, or several children of the same parents.

Whether or not the cases of splenomegalic cirrhosis described by Dr. Frederick Taylor in the Guy's Hospital Reports were examples of it or not is uncertain. Dr. Hayem and others have written accounts of several cases in France. The pathology of the affection is not yet fully understood, but there is a good deal of evidence to show that congenital syphilis is at the root of it. If this be so, it is very important that it should be fully realised, for the ultimate changes both in the spleen and in the liver are in the direction of fibrosis and cirrhosis. Fibrosis cannot be undone when once it has come about, but might well be minimised by the early adoption of anti-syphilitic treatment, the continued use both of mercury and iodides, and of such drugs as urotropine, which are so beneficial in catarrhal or infective lesions of the biliary passages.

We will give an account of an illustrative case from Dr. Hayem's collection. The patient was an only child. His mother and his mother's father suffered from the complaint, as will be described. As a child he had always seemed strong and well, but at fourteen he had some obscure abdominal attack, which resolved spontaneously at the end of three or four weeks, but was followed shortly afterwards by a series of convulsive seizures associated with loss of consciousness and biting of the tongue, but with no incontinence of urine. These epileptiform attacks recurred at intervals for three months, and then suddenly ceased, to recur no more. There was no personal history of venereal disease. At seventeen or eighteen years of age the patient gradually developed an icteric tint of his skin and conjunctivæ. This jaundice never disappeared again entirely. It was never extreme, though it varied a little from time to time, and it was still of about the same intensity twenty-eight years later.

For a long time he was able to attend to his work as a grain merchant, although hard physical exertion was beyond his powers. When twenty-one he had to undergo military service, and he served for four years in a cavalry regiment. Four times in those four years he was laid up in hospital with shortness of breath and palpitations, and an exacerbation in his jaundice, and severe attacks of epistaxis. The spleen was known to be enlarged even at that time, but on leaving the Army the man was able to do his ordinary work pretty well for some years. Notwithstanding the jaundice the appetite was good, there was no tendency to vomiting, and the stools were normally coloured with stercobilin. All sorts of remedies had been employed at various times—notably quinine, ferro-peptone, castor oil in big doses, magnesia, Hunyadi János, and calomel.

In 1897, when he was thirty-seven or thereabouts he became so troubled by pains in his left side, especially when lying on that side in bed, that he had to desist from active work; he lived a life of ease thereafter—and he is still alive. There has been no great change in his condition during the last ten years. The spleen is firm, smooth, and enlarged sufficiently to reach almost to the umbilicus. The liver is also firm, smooth, free from lumps, and enlarged so as to extend several fingers' breadths below the costal margin. There is a decided tinge of jaundice coupled with moderate anæmia, as indicated by the blood-count.

His mother is alive, aged 63, in excellent health, in spite of a hard-working life. She has neither brother nor sister, and she has had no other child than the patient. She was married at twenty-three, and she has had no particular illness except small-pox. Notwithstanding her uniformly good health, she has been jaundiced as long as she can remember. She is certain that she was yellow like she is now when she was a schoolgirl, and her playmates used to make fun of the colour of her skin and eyeballs. The jaundice is associated with no troublesome symptoms whatever. It is always present, but it varies in intensity, being very apt to increase after any work that is more fatiguing than usual. Physical examination reveals no abnormality of any organ except the spleen, and that, though decidedly less large than is her son's, is distinctly palpable below the costal margin. It should be mentioned that neither the mother nor the son were addicted to drinking too much alcohol.

The mother's father—*i.e.* the patient's maternal grandfather—developed jaundice when he was about twenty years of age, and this jaundice persisted with occasional exacerbations lasting a few days at a time. It is probably a point of importance that this grandfather had ulcerated legs: the ulcers started when he was eighteen. They were entirely cured for a time by treatment at a hospital, and later they recurred once more, to persist until his death.

There is no absolute proof that these ulcers were syphilitic, but beginning as they did at eighteen, it is unlikely that they were "varicose"; moreover, they were undoubtedly multiple. The patient himself—the grandson—presents no stigmata of syphilis. It is possible, however, that the pseudo-epilepsy of his youth was due to congenital syphilis. Dr. Hayem has found specific choroido-retinitis in some of his other cases of hereditary splenomegaly with chronic jaundice; and although the hereditary syphilitic suggestion is far from proved, it merits consideration as a guide both to the pathology and to the treatment of the condition.

